# Conversations in Cochlear Implantation: The Inner Ear Therapy of Today

**DOI:** 10.3390/biom12050649

**Published:** 2022-04-29

**Authors:** Grant Rauterkus, Anne K. Maxwell, Jacob B. Kahane, Jennifer J. Lentz, Moises A. Arriaga

**Affiliations:** 1 Tulane University School of Medicine, New Orleans, LA 70112, USA; grauterk@tulane.edu; 2 Department of Otorhinolaryngology and Biocommunications, Division of Neurotology, Louisiana State University Health Sciences Center, New Orleans, LA 70112, USA; amaxw3@lsuhsc.edu (A.K.M.); jkahan@lsuhsc.edu (J.B.K.); 3 Neuroscience Center of Excellence, Louisiana State University Health Sciences Center, New Orleans, LA 70112, USA; 4 Hearing and Balance Center, Our Lady of the Lake Regional Medical Center, Baton Rouge, LA 70808, USA; 5 Hearing Balance Center, Culicchia Neurological Clinic, New Orleans, LA 70112, USA

**Keywords:** hearing loss, cochlear implantation

## Abstract

As biomolecular approaches for hearing restoration in profound sensorineural hearing loss evolve, they will be applied in conjunction with or instead of cochlear implants. An understanding of the current state-of-the-art of this technology, including its advantages, disadvantages, and its potential for delivering and interacting with biomolecular hearing restoration approaches, is helpful for designing modern hearing-restoration strategies. Cochlear implants (CI) have evolved over the last four decades to restore hearing more effectively, in more people, with diverse indications. This evolution has been driven by advances in technology, surgery, and healthcare delivery. Here, we offer a practical treatise on the state of cochlear implantation directed towards developing the next generation of inner ear therapeutics. We aim to capture and distill conversations ongoing in CI research, development, and clinical management. In this review, we discuss successes and physiological constraints of hearing with an implant, common surgical approaches and electrode arrays, new indications and outcome measures for implantation, and barriers to CI utilization. Additionally, we compare cochlear implantation with biomolecular and pharmacological approaches, consider strategies to combine these approaches, and identify unmet medical needs with cochlear implants. The strengths and weaknesses of modern implantation highlighted here can mark opportunities for continued progress or improvement in the design and delivery of the next generation of inner ear therapeutics.

## 1. Introduction

Cochlear implants (CIs) are prostheses that electrically stimulate the cochlear nerve to restore not only sound perception, but speech understanding in people with profound sensorineural hearing loss. CIs use a battery-powered sound processor worn at ear level to transmit electrical signals to an electrode array that has been surgically implanted in the inner ear. The first generation of implants was approved by the FDA in 1984. These devices used a single electrode that allowed recipients to perceive the presence or absence of sound, while variably restoring some speech understanding [1,2,3]. The FDA approved the first multi-channel implants for adults and children in 1987 and 1990, respectively. 

Patients who undergo implantation today do so under a growing number of indications and use devices with a tonotopic array of as many as 24 electrodes. These modern CIs promote language acquisition, literacy, and academic performance in pre-lingually deaf children [4,5,6], while restoring meaningful speech recognition and generating better quality-of-life outcomes for adults who are unable to use traditional amplification [7,8,9].

While CI devices are a successful treatment option for many hearing-impaired individuals, several challenges related to their delivery, use, and access remain. Overcoming these challenges has fueled the investigation and development of biomolecular and pharmacologic therapeutic approaches using gene augmentation, gene-editing, antisense, and other small molecules [10,11,12,13,14,15,16,17,18,19,20,21,22,23]. Both approaches—CI devices and biomolecular/pharmacological drugs—target the inner ear to improve peripheral function and restore hearing. The CI circumvents defective or absent auditory hair cells to electronically stimulate a subset of spiral ganglion neurons or the nerve fibers of auditory neurons. In contrast, gene and antisense therapies are designed to target defective auditory hair cells directly to restore their function. Recent advances in the design of viral vectors used to deliver gene therapies and the expanding list of chemical modifications made to antisense oligonucleotides have significantly improved the cellular uptake of these drugs, thus demonstrating their potential to reach and treat nearly all inner and outer hair cells for more effective hearing outcomes [23,24,25,26,27,28,29,30,31,32,33,34,35,36,37,38,39]. 

As these new therapies continue their development and optimization towards translation into hearing impaired patients, we review the current clinical management with CIs. Here, we describe hearing with an implant and detail common surgical approaches and electrode arrays, new indications and outcome measures for implantation, and barriers to CI utilization. Finally, we discuss unmet medical needs for individuals being treated with CIs, and the opportunities for improvement with biomolecular and pharmacological approaches.

## 2. Hearing with an Implant

CIs are designed to restore speech perception for people with sensorineural hearing loss. They do not, however, replicate our hearing apparatus. Instead, CIs meet their purpose by layering an array of discrete electrodes covering the frequency range of human speech into the tonotopic infrastructure upon which we also rely to localize sound and hear music. Unsurprisingly, the extent to which CIs can process complex stimuli such as these is limited.

CIs cover a narrower range of frequencies than the cochlea (200–8500 Hz vs. 20–20,000 Hz), and they do so less accurately. Individual electrodes stimulate broad swaths of territory along the basilar membrane, often falling short of the apical turn [40,41]. As a result, CIs effectively collapse unique signals in a phenomenon called current spread [42]. Due to anatomical variations, such as pathological changes to the hair cells and spiral ganglion cells causing the patient’s hearing loss, and the limits of any given manufacturer’s device [42], electrodes are commonly misaligned with the cochlea’s natural frequency gradient [43]. This place–pitch mismatch underlies characteristic pitch perception deficits among CI users [42,44,45]. 

CIs also struggle to encode the temporal cues we use to perceive pitch in music, localize sounds, and hear speech in background noise. CIs cannot phase lock as our cochlea does [42]. Moreover, the basilar membrane can process both the gross waveform of a stimulus, as well as the more rapidly oscillating fine structure upon which it is carried. Historically, CIs have extracted that gross waveform, or the temporal envelope information from stimuli, and presented it in non-simultaneous pulses [46]. This process, called continuous interleaved sampling (CIS), can transmit enough information for a user to understand speech [47,48]. In accomplishing that task while preventing electrodes from distorting the activity of others, implants with enveloped-based strategies such as CIS discard a signal’s temporal fine structure processing (FSP) information altogether [42,49]. 

Along with deciphering speech from background noise, it is with this FSP information that we detect the lower-frequency, bass components of music [50]. A CI user relying exclusively on envelope information is unable to distinguish between samples of music with varying levels of bass. As much as 400 Hz of bass can be removed from musical stimuli before CI users recognize a difference [51]. This performance data pairs with the subjective finding that CI users do not often enjoy listening to music after implantation [52,53,54]. When they do, CI users tend to prefer less complex music with a clear beat and simple lyrics. Users also prefer music to which they were familiar prior to the onset of their deafness. 

Novel brain-imaging and sound-processing techniques have allowed us to identify cochlear stimulation and auditory training strategies that may improve music appreciation among this population [55,56,57,58,59]. Some manufacturers have even created and marketed devices that theoretically leverage FSP strategies to allow users to enjoy music and hear better in background noise. While these devices afford clinicians and patients the opportunity to exercise more choice in their hearing care, blinded paired comparisons of implants using both strategies do not consistently show FSP strategies to be superior to CIS in conserving music sound quality or speech recognition [60]. 

In addition to FSP, there are a variety of other strategies designed to improve the sound quality a CI user can experience when listening to music by stimulating more territory along the basilar membrane. Measuring the extent to which a stimulus must be altered to generate perceived differences in sound quality among CI users [51], research has shown that stimulation toward the apex via longer electrode arrays and bipolar stimulation that creates “phantom” channels beyond the physical boundaries of an array improves music sound quality perception [61,62]. 

## 3. Current Surgical Approaches to Implantation into the Cochlea

Modern cochlear implantation is a relatively routine and safe procedure. Nonetheless, operations can generate trauma, inflammation, fibrosis, obstructive hydrops, or synaptic changes in the inner ear that can manifest as residual hearing loss and vertigo [63,64,65,66,67]. 

For patients who have some residual hearing, threshold shifts are almost inevitable after implantation [68]. Today, modern technology and surgical techniques permit ‘softer’ approaches to implantation that can both treat a patient’s hearing loss and better preserve their residual hearing by protecting the structural integrity of the inner ear [67,69,70]. Early success with hybrid devices placed via the round window (RW) approach have encouraged clinicians and scientists to continue pursuing new and minimally invasive operative techniques [40,68]. Robot-assisted operations informed by advanced imaging that implant steerable [71,72,73], drug eluting devices may become the standard in cochlear implantation [74]. Currently, there are just three active operating systems that deploy robotics to access the middle ear [75]. The surgical approach and hardware in an implant remain tangible, significant contributors to patient outcomes [67]. 

Electrode arrays are most commonly implanted via the transmastoid facial recess approach with RW or cochleostomy insertion [76]. Some centers avoid the facial recess by employing a ‘suprameatal’ technique [77]. The ‘soft’ surgical approaches to implantation pioneered at the end of the 20th century were oriented around neural preservation via the use of perioperative systemic and topical steroids, meticulous avoidance of bone dust and surgical debris entering the cochlea, and slow, gentle insertion of more delicate electrode arrays. Early hearing preservation ‘soft’ surgical techniques relied on cochleostomies [67,70]. Today, RW insertion is more common, and most surgeons use ‘soft’ surgery techniques in all cochlear implantation surgeries, regardless of the length of the electrode being placed or a patient’s residual hearing status [78]. To date, cochleostomies are still a comparatively unstandardized set of procedures that rely on loosely defined anatomical landmarks [78]. Just 10% of neurotologists prefer cochleostomy to round window or extended round window approaches for electrode placement [79]. Even fewer choose cochleostomy when a patient has residual hearing to preserve. 

The RW itself presents a reliable landmark for a surgeon placing an electrode array [78], and RW insertions are associated with lower rates of electrode misplacement than cochleostomies [80]. This anatomical reliability is paramount given the considerable variability in ideal insertion vectors among different patients [81]. Computed tomography (CT) data indicate that RW insertions can place electrodes closer to the modiolus, and thus the spiral ganglion cells in the cochlea’s basal turn [76]. It is hypothesized that closer placement could mitigate current spread and generate better speech comprehension for the patient. Still, when electrodes are placed successfully, neither approach consistently results in better postoperative outcomes [80,82,83]. 

RW approaches are perhaps especially suited to placing shorter electrode arrays, such as those used for patients with substantial residual low frequency hearing. They are doubly favorable here, as histopathologic evidence indicates cochleostomies can seed an ossification process causing endolymphatic hydrops that characteristically costs a patient the low frequency hearing the operation aims to preserve, while treating their hearing loss [63]. These findings are consistent with others that demonstrate that the RW approach may be less traumatic [84], but trials comparing the approaches remain underway [85]. 

The design of a CI’s electrode array also impacts hearing preservation after implantation. The two major categories of array are straight lateral wall (LW) and curved peri-modiolar (PM) [40]. PM arrays are curved to closely hug the modiolus along the medial wall of the cochlea; however, this perimodiolar position may mitigate interference between electrodes by directly stimulating spiral ganglion cells [40,67]. LW arrays lie some distance farther from the modiolus and must stimulate the nerve fibers of the auditory neurons, as opposed to the neurons themselves. Accepting potentially more crosstalk between electrodes but limiting trauma with the delicate structures of the inner ear, LW arrays are preferred for hearing preservation in hybrid implant candidates. 

Pure hybrid implants have electrodes that are roughly a third the length of typical arrays. There are also longer, short-LW arrays that offer slightly more coverage in the cochlea, while appearing to preserve hearing in the lower frequencies [86], though not at the rates of the truly short electrodes. Currently, many surgeons prefer to implant patients with longer electrodes even if they meet criteria for a hybrid implant [79]. Longer electrodes are thinner than ever before, and with modern surgical techniques, they can allow for the preservation of a patient’s residual hearing while covering more of the cochlea. Patients with even substantial residual low frequency hearing at the time of implantation can lose it as their underlying hearing loss progresses, or because of surgical sequelae such as that of cochlear fibrosis or endolymphatic hydrops [63]. If a patient who was originally implanted with a short electrode loses their residual hearing, they may need to undergo re-implantation with a longer electrode. Revisions and re-implantations are notoriously challenging. 

The growing use of cone beam CT imaging has allowed for intraoperative and postoperative evaluation of electrode placement. CT scans can show electrode dislocation, tip fold-over, and mispositioning. This allows for real time visualization of the electrode and revision of the insertion at the time of initial surgery [87]. When combined with expected electrical distribution of charge from an electrode, postoperative cone beam CT facilitates the deactivation of interfering electrodes, which can improve speech recognition [88,89,90]. 

## 4. Growing List of Indications for CIs

### 4.1. Single-Sided Deafness

Bone-conduction hearing aids (BCHA) and contralateral routing of signals (CROS) hearing aids have long been employed for patients with profound unilateral hearing losses (UHL) who pursue care [91]. These devices deliver signals from the side of the user’s hearing loss to the hearing ear, without restoring the binaural signals that contribute to sound localization or hearing speech in complex listening conditions. 

CIs present a means of restoring these binaural cues in patients with UHL, and in 2019, the Food and Drug Administration (FDA) expanded CI indications to include “patients 5 years and older with single sided deafness (SSD) and asymmetric hearing loss (AHL) who have profound sensorineural hearing loss in the ear to be implanted and normal hearing or mild to moderate sensorineural hearing loss in the other ear” [92]. 

The approval followed the proliferation of evidence that adults with acquired UHL can access binaural hearing benefits in localization and masked speech perception, sometimes within months of implantation [93,94,95]. In their approval, the FDA recommended against implanting patients with UHL who have had a profound loss for more than a decade. While duration of deafness negatively predicts speech perception performance in patients who undergo implantation for bilateral losses [96], data suggest this trend may be less predictive in adults who undergo implantation for SSD [97]. 

### 4.2. Tinnitus

Some of the data that contributed to the approval of CIs for SSD came from studies of implantation as a treatment for tinnitus [98,99,100]. Most patients with sensorineural hearing loss experience tinnitus, with varying levels of associated handicap [101,102,103]. The novelty in the first study of implantation in patients with UHL and tinnitus was that patients presented to the clinic for tinnitus, and not their hearing loss [104]. While tinnitus is still not itself an indication for cochlear implantation, the condition can be profoundly debilitating. 

Studies in the SSD setting were initiated by the observation that tinnitus suppression was a common side effect of treatment among people who underwent implantation for bilateral hearing losses [103]. These trials were not powered to evaluate tinnitus suppression as a primary outcome. Tinnitus suppression was an incidental, albeit positive finding for varying numbers of patients [105]. Moreover, the positive effect appears to persist in some patients with UHL who underwent implantation driven by tinnitus [102,106,107,108]. 

SSD trials of implantation for tinnitus have their own limitations. Patients can be neither randomized nor blinded. The most ubiquitous outcome measures for tinnitus [109], while not specifically designed to move with treatment, can be pooled. In their systematic review, Peter et al. reported that more than 80% of patients with SSD and tinnitus experienced complete suppression or a decreased burden after implantation [102]. Unfortunately, 5% of patients’ tinnitus worsened after treatment. The mechanism for either of these responses to implantation remains as unclear as that for the pathophysiology of tinnitus itself [110]. Still, these results have created interest in analogous implantable treatments for people who have incapacitating tinnitus but do not qualify for CIs [111].

Patients who already use a CI and experience tinnitus rely on a combination of patient education, sound retraining therapy, and cognitive behavioral therapy, which are common strategies for other patients reporting tinnitus [112]. While hearing aid users can take advantage of sound therapy tools programmed into their devices, CI users must use external sources. 

### 4.3. Hybrid Implants

Bilateral profound hearing loss in the high frequencies prevents people from understanding speech, especially in noisy conditions, even if their low frequency hearing remains intact. Until recently, patients with profound high frequency losses and significant residual low frequency hearing were left to contend with the most socially isolating outcomes of their loss, without a solution [113]. Importantly, the etiologies of ski-sloping high frequency losses commonly disable the inner and outer hair cells, precluding meaningful improvement in speech comprehension via traditional amplification [114]. 

In 2014, the FDA approved a hybrid device with the function of a hearing aid and a cochlear implant with a shorter electrode array that stimulates just the high frequency, basal turn of the cochlea [115]. These devices lessen the risk to the user’s residual hearing, as they do not encroach on the part of the cochlea responsible for the lower frequencies. Impressively, they also do not prevent acoustic waves from traveling the length of the cochlea [114]. 

By the time of their approval, these devices were not themselves new [114,116]. However, the approval of hybrid CIs marked a major development in the criteria for CI candidacy at large [117]. Patients with steeply sloping high frequency losses and intact low-frequency hearing could become eligible for implantation outside of the standard full-sentence recognition testing in their best aided condition. Candidacy for these devices was established with aided, single-word recognition, a task much more sensitive to the practical implications of a high frequency loss [118,119]. 

Experience with these devices has demonstrated that people can integrate acoustic and electrical signals, and hybrid implants effectively restore the consonant recognition necessary for users to unlock their residual lower frequency hearing [120,121,122,123]. Patients outperform their preoperative baselines in monosyllabic word recognition [113,120]. More directly due to the synergy between their electrical hearing and their residual acoustic hearing, hybrid CI users tolerate a lower signal-to-noise ratio than traditional CI users when listening to speech presented in background noise [113]. The preservation of lower-frequency hearing also helps hybrid CI users outperform traditional CI users in music-recognition tasks, both with and without lyrics [122]. 

Prolonged hearing preservation is possible among hybrid CI users; there is evidence of significant preservation 15 years after implantation [124]. Nonetheless, we do not yet understand the factors that contribute to varying levels of preservation among different people [67]. Gender and device type do not appear to contribute to differing outcomes, while age at implantation can [125]. There are mixed reports for whether and how preoperative hearing predicts postoperative preservation [86,125]. 

Despite the efficacy of hybrid implants and the potential for patients to integrate acoustic and electric signals in the same ear, a host of case series, cohort studies, and retrospective reviews have reported patients who initially use hybrid devices frequently move to rely solely on electrical stimulation [126,127,128,129]. Some of these patients rely on electrical stimulation because they have lost their residual hearing over time, but others actively chose not to continue using electro-acoustic stimulation. Discomfort and poor sound quality are reported to drive some of these patients’ decisions, while aesthetic concerns and physical comfort drive others. Additionally, given the option, some patients prefer not to wear devices that extend into the ear canal. 

### 4.4. Cochlear Nerve Pathology

Auditory brainstem implants (ABI) are analogous to CIs, save that their electrodes are on a flat mesh rather than a wire array, and they stimulate the cochlear nucleus in the lateral recess of the fourth ventricle in the brainstem. These devices were pioneered for patients with Neurofibromatosis type 2 (NF2), where they are used after bilateral acoustic neuromas or treatments thereof cause profound hearing loss [130]. Unfortunately, these prostheses resemble the very first generation of single electrode CIs. ABIs can reliably restore sound awareness that can contribute to environmental safety and lip reading, but they are limited in their ability to restore speech comprehension [131,132]. 

In managing NF2, physicians take every measure to preserve the cochlear nerve to the extent that the patient could still benefit from a CI, rather than an ABI. Surgeons have simultaneously resected acoustic neuromas while placing CIs for 30 years [133]. Until 2012, however, there were fewer than three dozen such reports in the literature [131]. As evidence has accumulated, we have only become more confident that there is a subgroup of patients with acoustic neuromas whose benefit from CI use is commensurate with patients who undergo implantation under other indications [131,134,135]. 

Unlike NF2, which causes tumor-related cochlear nerve loss, cochlear nerve aplasia is a potentially treatable cause of deafness in children. Colletti et al. have consistently reported excellent results and even open set discrimination in children who use ABIs for cochlear nerve aplasia [136]. 

### 4.5. Older Adults 

Hearing loss is a significant, but modifiable risk factor for cognitive decline [137]. Hearing loss also presents a major barrier to healthcare access and utilization [138], while putting older adults at increased risk of falling and mental health issues, such as depression and anxiety [139,140]. 

Naturally, CIs are being used and studied in older adults with hearing loss with increased frequency. Trials of older adults with and without cognitive impairment undergoing implantation report that the devices are safe to use and improve patients’ speech understanding and quality of life [141,142]. While the effect of cochlear implantation on cognition in older adults remains under investigation, trials have demonstrated improved executive function in a subset of users [8]. Although some data suggests patients older than 80 years of age do not benefit from implantation the same way younger adults do [143], this finding may be mediated, in part, by differences in the frequency with which patients use their implants [144]. 

Critical to the ongoing use of these devices in older populations is the finding that age and overall health do not appear to appreciably change a patient’s risk for CI-related complications [145]. Nonetheless, a patient’s unique comorbidities and risk profile must factor into their CI candidacy assessment. For patients who are less suited to undergo general anesthesia, for instance, local anesthesia protocols are safe and effective when deployed by experienced clinicians in tertiary care centers [146]. Patient motivation is also an important consideration in candidacy for older adults. After implantation, people who are predisposed to perform poorly with an implant are commonly recommended for auditory training that supplements their exposure to complex sounds [55]. Several of the manufactures offer their own programs. This practice mirrors the relatively more intense auditory training and speech language therapy that is standard for pre-lingually deaf children who use CIs. 

## 5. Outcome Measures

The success of a CI is typically evaluated based on a user’s ability to recognize speech in quiet and noisy conditions. However, these performance indicators do not reflect users’ self-perceived communication abilities or the CI’s impact on their quality of life [147]. Previously, hearing-specific and CI-specific patient reported outcome measures showed significant improvements in quality of life, driven by implantation [148]. The mismatch between patient-reported outcome measures and performance evaluations has guided the recent development of the CIQOL-35 and CIQOL-10 Global, both of which are practical tools that measure quality of life, specifically among CI users, in clinical and research settings [149,150]. 

The arrival of these instruments is well-timed. They are the most psychometrically sound instruments we have for CI users and can therefore help clinicians interrogate the real-world value of ongoing iterative changes in implantation techniques and technology [149,151]. Given the countless inner ear therapeutics on the horizon for several subgroups of people who are eligible for cochlear implantation, these instruments may also guide the patient-reported outcome measures that will necessarily drive the regulatory approval of a potential pharmacological alternative for a given disease state [152].

### Genetics and Outcomes

Investigations into patients who do not receive the expected benefit from cochlear implantation have expanded our understanding of how genetic factors influence auditory outcomes. Notably, CI recipients with genetic derangements of spiral ganglion function perform lower on speech perception tests than patients whose mutations disrupt the organ of Corti [153]. Miyagawa et al. demonstrated that the majority of prelingually implanted children with nonsyndromic hearing loss and specific deafness gene mutations demonstrate good CI outcomes and rapidly develop auditory skills, while those with syndromic hearing loss or inner ear and/or cochlear nerve malformations exhibit moderate–poor CI outcomes [154]. Additionally, identifying genetic etiologies has implications for hearing preservation with CI. Yoshimura et al. identified three causative gene mutations only expressed in hair cell stereocilia that permitted improved hearing preservation outcomes, compared with other genetic disruptors of cochlear function [155]. A recent large-cohort study of 459 CI recipients identified causative genetic mutations in 48% of children and 22% of adults, establishing a foundation for future studies correlating genetic cause with CI performance [156].

## 6. Barriers to Care

### 6.1. Navigating Implantation

In a 2018 survey of 81 neurotologists, 78% reported that they had performed an off-label implantation in the previous 2 years [157]. Respondents at higher volume, academic centers perform implantations under these off-label indications most frequently, but they are not alone. These cases generate data and experience that is eventually reflected in expanding FDA labeling on new and pre-existing devices, but CI indications remain years behind practice. 

The FDA approved a device for children starting at 9 months of age for the first time in 2020 [158,159]. This most ambitious approval does not adhere to the latest 1-2-3 standard set by the Joint Committee on Infant Hearing (JCIH), wherein children with congenital hearing losses are identified at their newborn hearing screening, evaluated in their second month, and outfitted with an appropriate intervention in their third. Children who will ultimately undergo implantation are often fitted with a hearing aid in their third month. The 9-month figure from the FDA accounts for time during which a child who is identified with hearing loss could attempt to use hearing aids, failure with which is a prerequisite for implantation [157]. This failure criterion is a vestige of adult CI candidacy standards. Evidence from several studies shows that surgery should not be delayed to account for a lengthy amplification trial in children, especially when bilaterally absent auditory brainstem responses have been confirmed with behavioral audiometric testing [160,161,162]. Even and perhaps especially for children on this ‘fast-track,’ skull and mastoid size, auditory and neurological maturation, and risks of anesthesia exposure must inform the timing of cochlear implantation. Regulatory and practical factors force clinicians and families to navigate their own, off-label timeline to safely guarantee children the earliest opportunity to develop aural communication.

### 6.2. Cost

While regulatory approvals and off-label uses for CIs proliferate, an insidious set of socioeconomic barriers also prevents patients in need of accessing implantation. In the United States (US), it is estimated that less than 10% of adult and 50% of pediatric candidates utilize CIs [163,164,165]. Moreover, adults who ultimately become users can experience wait times of more than a decade [98,163,166]. Importantly, duration of deafness is one of the strongest predictors of poor performance after implantation [97,167]. CI candidates who identify as nonwhite, who are already more likely to have poorer preoperative word recognition scores than their peers, wait the longest for implantation [163,168,169]. This disparity broadens when we account for the lower likelihood that nonwhite CI candidates undergo implantation at all [169]. 

In the US, children and adults accessing their care via Medicaid who qualify for CI candidacy do not experience the same outcomes as those who are covered by private payers [170,171]. This is to be expected; for adults, Medicaid coverage for cochlear implantation is determined by individual states, and more than a third of them do not cover implantation at all [172,173]. Medicaid beneficiaries also struggle with post-implant complications and access to critical follow up care due to multiple fiscal and logistical barriers [170,171,172], as families with fewer resources access fewer healthcare resources, even if they are ostensibly subsidized [174]. 

The care pathway for patients with private insurance is different, but not necessarily more straightforward. Private payers commonly disregard not only published scientific evidence, but also clinical practice guidelines, and inconsistently acknowledge common CI indications. It is not uncommon for a private payer to refuse to cover implantations for children under a year of age and those with SSD [175]. 

## 7. Technology in Development

### 7.1. Optical Cochlear Implants

Basic research on the feasibility of an Optical Cochlear Implant (oCI) using photonic stimulation of the hair cells or spiral ganglion cells rather than electrical current as used in the current Electrical Cochlear Implant (eCI) suggests a theoretical possibility of improving the dynamic range of current eCI stimulation strategies (which could enhance understanding in background sound and music appreciation) and more focused neural stimulation than eCI (which could limit electrode “cross talk”). Recent reviews provide an excellent summary of the major issues related to oCI [176,177]. 

Two basic strategies are under investigation: Infrared Neural Stimulation (INS), which encodes sound by creating heat with an implanted laser to initiate neural stimulation; and Optogenetic Stimulation, which expresses photosensitive ion channels to neurons. Despite the potential, investigators have several significant obstacles to overcome. With INS, the challenge of balancing the heat generation to create enough to stimulate without damaging the cells is formidable. There is still controversy as to whether or not such INS stimulation is producing direct neural stimulation from the localized thermal effect or if there is an optoacoustic event stimulating surviving neurons from the stress-relaxation waves following confined heating within the cochlea in animal experiments [176]. With Optogenetic Stimulation, the blue–green stimulation of light-sensitive ion channels such as Chronos-mediated stimulation risked phototoxic damage to cells and the newer strategies emphasize red-shifted stimulation with ChrimsonR which avoids the ototoxicity and offers improved firing rates in experimental designs. These optogenetic strategies rely on viral gene transfer with Adeno-associated Viruses as the main candidate for future application. Such optogenetic strategies open the possibility of Active oCI and Passive oCI stimulation with either implantation of a micro-LED (light-emitting diode) arrays versus passive waveguide-based implantation with emitter arrays spread through the area of implantation [177]. However, there are still formidable challenges in designing such arrays for safe implantation, such as understanding the neural effects of prolonged stimulation, current requirements, and durability. While the concept of oCI merits further investigation, clinical application of an oCI is not imminent.

### 7.2. Electrode Coating and Drug Elution

The placement of the CI electrode array within the scala tympani necessarily disrupts the microenvironment of that delicate inner ear structure. A silicone carrier delivers the electrodes from the receiver–stimulator to the cochlea. Recent developments have allowed special grafting and coating of materials onto the silicone electrode carrier. Materials which reduce friction and insertion trauma have already been implemented in animal models with some success [178]. The preservation of acoustic hearing in the setting of cochlear implantation will likely be facilitated by further developments and improvements in electrode delivery. 

Development of coating materials not only allows for atraumatic electrode insertion, but may also enable the delivery of drugs and other biologically active compounds directly to the inner ear. Animal studies evaluating the safety of steroid-eluting electrode arrays are well underway. The ability to deliver steroids to the scala tympani provides an exciting opportunity to further advance acoustic hearing preservation and reduce vertigo in the setting of cochlear implantation [179]. Finally, research teams are investigating the use of biologically active particles grafted to the electrode that would allow the on-growth of new spiral ganglion cells within the inner ear [180].

### 7.3. Intraoperative Monitoring

Intraoperative facial nerve monitoring has long been the practice for CI surgeons to preserve facial motor function during the delicate surgical procedure. Within the last decade, there have been significant developments of additional intraoperative monitoring procedures to evaluate the electrode insertion process and final placement prior to closure. Impedance measurements and neural response telemetry can be obtained after electrode placement and prior to closure to partially evaluate device integrity and placement.

To further preserve acoustic hearing in patients undergoing CI, new intraoperative monitoring techniques have been developed to evaluate cochlear trauma and direct the surgeon to more gentle insertion. While a full discussion of these techniques is beyond the scope of this manuscript, intraoperative electrocochleography measures electrical potentials generated within the cochlea and can be used to evaluate preservation of function during insertion. The results of large reviews on the efficacy of this type of monitoring in the clinical setting are largely mixed [181]. More recently, the use of transimpedance matrices allows for the detection of tip fold over and fine details of electrode positioning, such as proximity to the lateral wall [182,183]. These intraoperative monitoring techniques are not yet in wide clinical use but may prove to be a useful adjunct for plain-film X-ray prior to wound closure.

## 8. Cultural Considerations 

There is a heterogeneous community of people who understand deafness not as a disability, but as a cultural characteristic to be embraced as part of an individual’s identity. Voices from this community are a constant and impactful presence in conversations that establish the goals of early childhood interventions for hearing loss, deaf education, and the experience of deafness. 

We are not attempting to write on behalf of the people in this community, nor to summarize what is a lived-in spectrum of deeply held beliefs about cochlear implantation. Similarly, we do not speculate as to their positions about forthcoming inner ear therapeutics. Our purpose here is to call attention to a constituency who will be a part of the coming conversations about interventions making their way into and beyond clinical trials. 

## 9. Conclusions

Themes from the conversations that define the CI space can inform the development and evaluation of an oncoming wave of inner ear therapeutics. CIs are the standard we have in treating so many of the conditions for which there are inner ear therapeutics in pre-clinical and clinical pipelines [183]. Understanding the physiological and practical constraints of implantation can help us evaluate not only the efficacy of forthcoming therapies, but also their distribution. 

A small molecule, gene, or antisense therapy designed to preserve or restore hearing could avert surgical implantation entirely, or postpone it. Alternatively, if these treatments selectively restore hearing in some frequencies, they may create a novel use case for combination therapy. Preclinical studies using gene and antisense therapies show impressive rescue of low- and mid-frequency sensitivity, demonstrating that these drugs can reach the cochlear apex [17,25,26,27,29,30,31,32,33,37,38,39], which is currently not accessible with most implantation techniques. Additionally, CIs could be designed to be a drug-delivery conduit for biomolecular or pharmacologic drugs using drug-eluting strategies or creating ports for drug delivery within the cochlea. The optogenetic strategies used in oCIs under investigation are a prototype of combining inner ear drugs with cochlear implantation and highlight the importance of considering the future of treating the inner ear with a combined biomolecular and biodevice approach. 

This review features the ongoing improvements in CIs and the major shortcomings in CI outcomes for listening in background sound, music appreciation, surgical and post-surgical complications, and the age of implantation limiting the therapeutic window that highlight the unmet medical needs for individuals with a hearing impairment. Gene and antisense therapies aim to reach all defective hair cells along the basilar membrane and, in doing so, may improve hearing in noisy environments and the appreciation for music. Several delivery routes to the inner ear have been investigated, including topical-tympanic membrane application (antisense), trans-tympanic injection (small molecule and antisense), intracochlear injection via RW injection or cochleostomy (gene and antisense), and intralabyrinthine injection via the utricle or canalostomy [13,26]. The less-invasive surgical injection of these drugs offers the opportunity for earlier treatment in infancy when significant neurocognitive development depends on sensory input. Older adults who are not a candidate for the more extensive surgery required for implantation may be a candidate for an inner ear drug that uses a less invasive surgical delivery. 

Recipients of new inner ear therapeutics may still benefit from the speech and auditory training that have been honed over decades with CI users. As we have learned with cochlear implantation, rehabilitation and hearing therapy is a significant component of therapeutic success beyond just providing sensory input. Patients pursuing new therapies will likely need to confront a set of economic and social barriers that overlaps those facing CI candidates. These and other tensions encountered over decades of implantation and use can direct opportunities to address unmet medical needs as we develop a new standard of care for people with hearing loss. 

## Data Availability

Not applicable.
